# An Approach for Diagnosing and Treating Neurosyphilis: A Case Report

**DOI:** 10.7759/cureus.19631

**Published:** 2021-11-16

**Authors:** Spencer J Glenn, Saba Haq, Keshav Poddar, Leigh Hunter

**Affiliations:** 1 Internal Medicine, Methodist Dallas Medical Center, Dallas, USA; 2 Internal Medicine, Methodist Health System, Dallas, USA; 3 Infectious Disease, Methodist Health System, Dallas, USA

**Keywords:** paresis., paresthesia, cerebrospinal fluid, tabes dorsalis, syphilis, treponema pallidum, neurosyphilis

## Abstract

Neurosyphilis is a disease caused by systemic infection with *Treponema pallidum*, which infiltrates the central nervous system and preganglionic dorsal roots. This process presents as neurological deficits and can occur any time during the infection course, but usually takes many years. Neurosyphilis is rare in the developed world where antibiotics are readily available to treat the early stages of syphilis. This report describes a case of neurosyphilis in a 71-year-old woman who presented with ataxia and vision changes and was ultimately found to have a positive rapid plasma reagin test and protein in the cerebrospinal fluid. She was treated with intravenous penicillin for two weeks with a good response.

## Introduction

Syphilis is a bacterial infection typically caused by sexual contact. The number of syphilis cases has been slowly increasing since the early 2000s and increased 74% since 2015 [1]. In 2019, there were 129,813 reported cases of syphilis of all stages. Luckily, syphilis responds to antibiotic therapy, and rates of overall syphilis declined significantly with the advent of penicillin.

Neurosyphilis is an infection of the central nervous system usually in patients with untreated syphilis. It is uncommon since earlier stages of syphilis are usually recognized and treated. Neurosyphilis presentations may include stroke-like symptoms, meningeal symptoms, or paresis. The patient may have visual or hearing loss, be asymptomatic with abnormal cerebrospinal fluid (CSF) labs or present with classic findings of tabes dorsalis with sensory ataxia [2]. We report the case of a patient ultimately diagnosed with neurosyphilis who presented with ataxia and vision changes. This report is to raise awareness of a now uncommon and often difficult to recognize presentation and provide knowledge of appropriate treatment.

## Case presentation

A 71-year-old African American female with a medical history of hypertension, type 2 diabetes mellitus, stage 3 chronic kidney disease, and osteoarthritis initially presented to the emergency room with intermittent bilateral hand tingling and numbness, which was gradual in onset over months. Her symptoms were associated with seeing red spots and experiencing a burning sensation in the bottom of her feet. She was concerned because she was having difficulty picking up objects due to her hand symptoms. Her basic lab work was unremarkable, and she was provided gabapentin and magnesium oxide with close follow-up with her primary care physician. At follow-up, she complained of one to two months of unsteady gait as well as increased confusion. Her reported medications included losartan and metformin. Physical exam revealed normal pupils with reaction and accommodation (3mm diameter bilaterally), no cranial nerve deficits, normal strength throughout, and normal reflexes throughout except for diminished reflexes in the bilateral knees and ankles. She endorsed blurry vision; no ophthalmologic exam was performed, but she was able to read a name badge from one foot away. She additionally was found to have decreased vibratory and proprioception in a stocking pattern as well as a wide-based and unsteady gait. 

To investigate further, tests for thyroid-stimulating hormone (TSH), folate, B12, and rapid plasma regain (RPR) titer were ordered. Her TSH, folate, and B12 levels were within normal limits, but her RPR titer was reactive at 1:1. A subsequent reflex *Treponema pallidum* particle agglutination (TP-PA) test was reactive. She was told to go to the hospital for further workup and treatment. Further questioning revealed that she had had two sexual partners in her life, both ex-husbands. However, she noted that her husbands committed adultery several times and that she was not currently sexually active; her last sexual encounter occurred several years ago. She endorsed that she did not notice any ulcers or skin lesions, and she had not been treated for syphilis. During hospitalization, she received a lumbar puncture that revealed a negative venereal disease research laboratory (VDRL) test result. Her glucose level (61 mg/dL) and white blood cell level (3 x 10^9^/L) were normal, but her CSF protein level was markedly elevated at 156 mg/dL. Due to her exposure history, a constellation of symptoms, RPR titer with positive TP-PA, and protein elevation in her CSF, our infectious disease colleagues believed that she contracted syphilis earlier in life from one of her husbands and was never treated. The decision was made to start treatment with intravenous (IV) penicillin (24 million units, continuous infusion) for two weeks. At follow-up, her vision had improved and she no longer had sensory symptoms in her hands. Her gait was somewhat improved, but still unsteady, and she still had burning pain in her feet. Her physical exam showed improvement in sensation in hands and improved gait; all other neurological exam was unchanged. It was thought that neuropathy in feet could have been due to poorly controlled diabetes; although, no formal nerve conduction study was pursued.

## Discussion

Syphilis is commonly transmitted via direct contact with lesions infected with *T. pallidum*, usually during sexual contact. Neurological manifestations occur when there is an infection of the CSF with the bacteria. Efficacy of treatment with penicillin has resulted in less disease prevalence, especially late manifestations of syphilis; IV penicillin remains the mainstay of neurosyphilis treatment. Guidelines for treatment are outlined based on several controlled trials, meta-analyses, and cohort studies [3].

Our patient was most likely exposed to syphilis many years ago from one of her husbands. She was never treated for syphilis to her knowledge, and most likely developed neurosyphilis from years of going untreated. Suspicion was raised given her cluster of symptoms: her visual changes, gait unsteadiness, and numbness in her hands. She likely had late neurosyphilis with evidence of tabes dorsalis. Typically, these patients will have paresthesia symptoms, shooting pains, ataxia, and visual symptoms. She did not have classic physical exam findings such as Argyll Robertson pupils, ocular palsies, or Charcot's joints; however, she did have decreased reflexes from the knee down as well as proprioception and sensory deficits in a stocking pattern. She did not exhibit signs of generalized paresis commonly seen in late syphilis; she had no dementia, mood lability, or tremors [4]. In addition, the initial workup showing normal levels of B12, folate, and TSH with a concomitant RPR of 1:1 raised the suspicion for syphilis.

TP-PA testing is 100% specific for the detection of syphilis; it is an excellent test for confirmation when the disease cannot be ruled out (Table 1) [5,6]. A positive TP-PA test prompted the next step in our patient’s diagnosis, which was to perform a lumbar puncture and test the CSF for evidence of syphilis. CSF VDRL test is currently recommended by the Centers for Disease Control for confirming neurosyphilis due to its very high specificity [6]. However, the CSF VDRL test is not sensitive; we could not rule out neurosyphilis due to a negative result. Protein in the CSF is neither sensitive nor specific for neurosyphilis; however, the elevation of protein has been associated with neurosyphilis and current recommendations support consideration of neurosyphilis in symptomatic patients with elevated protein or pleocytosis [7]. Currently, there is no gold-standard testing for neurosyphilis; diagnosis is based on clinical presentation and a combination of lab testing. Based on her neurological symptoms, positive RPR, confirmatory TP-PA, and elevated protein in the CSF, the decision was made to treat this patient for neurosyphilis with continuous IV penicillin for two weeks.

**Table 1:** Sensitivity and Specificity of Assays for the Detection of Syphilis Data extracted from meta-analyses conducted by Cantor et al. [5] and Park et al. [6]. Abbreviations: RPR, rapid plasma regain; VDRL, venereal disease research laboratory; TP-PA, Treponema pallidum particle agglutination

	Primary syphilis		Secondary syphilis		Latent Syphilis (Early)		Latent Syphilis (Late)	
	Sens.	Spec.	Sens.	Spec.	Sens.	Spec.	Sens.	Spec.
RPR	86%	98%	100%	98%	98%	98%	73%	98%
VDRL	78%	98%	100%	98%	96%	98%	71%	98%
TP-PA	94.5%	100%	100%	100%	100%	100%	86.8%	100%

## Conclusions

Neurosyphilis is rarely seen in the developed world due to the use of antibiotics to treat early syphilis infection. It is possible that this patient had tabes dorsalis, which is even rarer. Neurosyphilis symptoms can be non-specific, making it difficult to diagnose and treat. Furthermore, neurosyphilis often goes unrecognized because it is not seen in clinical practices as often as it once was. Many assays are available for the detection of syphilis and have varying levels of specificity and sensitivity. A thorough history and physical exam paired with laboratory testing of the serum and CSF can assist in the diagnosis of neurosyphilis. Neurosyphilis-associated morbidity can easily be treated with therapies when the diagnosis is made. The current case provides insight into common presentations of neurosyphilis and a guidelines-based approach to making a diagnosis. Further investigation of the diagnostic characteristics of different CSF assays may provide better guidelines to diagnosis neurosyphilis and further alleviate morbidity associated with this curable ailment.
